# Supplementary material for: An FPGA-based analysis of trade-offs in the presence of ill-conditioning and different precision levels in computations
Source: PLoS One. 2020 Jun 19;15(6):e0234293. doi: 10.1371/journal.pone.0234293 (PMC7304599; doi:10.1371/journal.pone.0234293)
Supplement: S1 File — (PDF) [file pone.0234293.s001.pdf]

Supporting material.

1 List of terms and simbols

1.1 List of terms:

In this section we show the list of simbols and terms used in the manuscript entitled: "Trade-off analysis in hardware architectures for the solution of systems of equations with ill-conditioned matrices and different precisions".

BRAM: Block Random Access Memory
DP: Double precision
DSP: Digital Signal Processor
ECG: Electrocardiography
EEG: Electroencephalography
EP: Extra precision
FF: Flip-flop
FPGA: Field Programmable Gate Array
HP: Half precision
IOB: Input/Output Block
LUT: Look-up Table
LUTRAM: Look Up Table Distribution RAM
MUX: Multiplexor
SP: Simple precision
VHDL: VHSIC (Very High Speed Integrated Circuit) Hardware Description.

1.2 List of simbols:

$\Omega = \overline{\Omega}_1 \cup \Omega_2$ is a bounded region sufficiently smooth of $\mathbb{R}^d$, with $d = 2$ or 3, which is considered as conductor media compound by two bounded regions Ω_1 and Ω_2, where Ω_2 is an annular region and $\Omega_1 = \Omega \setminus \overline{\Omega}_2$.

σ_1 and σ_1 denote the conductivities of regions Ω_1 and Ω_2, respectively.

$S_1 = \partial\Omega_1$ and $S_2 = \partial\Omega$ are the boundaries of Ω_1 and Ω, respectively.

g is a funtion that represents the source defined on the boundary S_1.

Δ represents the Laplace operator, which is also denoted by ∇^2.

u represents the electric potential in Ω, and $u_i = u|_{\Omega_i}$, $i = 1, 2$.

n_1 and n_2 are the unit outward normal vectors on the boundary of Ω_1 and Ω_2, respectively.

$\frac{\partial u_i}{\partial n_i}$ denotes the normal derivative of u_i in S_i with respect to the normal unitary vector n_i, which is exterior to Ω_i, $i = 1, 2$.

$L_2(\Omega_i)$, $L_2(S_i)$ and $L_2(\Omega_0)$ are the spaces of square integrable functions defined on Ω_i, S_i and $\Omega_0 = \overline{\Omega_1 \cup \Omega_2}$, respectively, $i = 1, 2$.

$H_1(\Omega_i)$, $i = 1, 2$, and $H_1(\Omega)$ denote the corresponding Sobolev spaces of the functions in $L_2(\Omega_i)$ and $L_2(S_i)$, respectively, whose generalized first derivatives are also square integrable functions.

$u|_{S_i}$ denotes the trace to S_i of the function $u \in H_1(\Omega_i)$ for $i = 1, 2$, corresponding to the restriction of a continuous function in the classical sense.

We use the subscript $\perp$ in the subspaces $L_2^{\perp}(\Omega_i)$ and $L_2^{\perp}(S_i)$ of $L_2(\Omega_i)$ and $L_2(S_i)$ to denote the spaces of functions that are orthogonal to the constants with respect to the corresponding scalar product, i.e., if W is any previous space then, $W_{\perp} = w \in W : \langle w, 1\rangle_W = 0$, where $\langle , \rangle_W$ is the scalar product of W.

$\| \cdot \|_W$ denotes the norm of the space W, which can be a normed or Hilbert space, such as $L_2(\Omega)$, and $H_1(\Omega)$, where Ω is a region of $\mathbb{R}^d$, for $d = 1, 2, 3, ...$

A^{-1} denotes the inverse matrix of a square matrix A.

$det(A)$ denotes the determinant of a square matrix A.

$adj(A)$ denotes the adjoint matrix of a square matrix A.

$cond(A)$ denotes the condition number of a square matrix A.

$AE(\cdot, \cdot)$ and $RE(\cdot, \cdot)$ denote the absolute and relative errors, respectively, regarding the Euclidean norm in $\mathbb{R}^2$.

2 Additional results

2.1 Additional tables for example 1

TRUNCATION ERRORS: In example 1 of the manuscript, we consider the matrix $A = \begin{pmatrix} 1 & 1 \\ 0 & \varepsilon \end{pmatrix}$, where ε takes different positive values less than 10^{-2}. In this case, $det(A) = \varepsilon$ and the inverse matrix of A is given by

$$A^{-1} = \frac{1}{det(A)} adj(A) = \frac{1}{\varepsilon} \begin{pmatrix} \varepsilon & -1 \\ 0 & 1 \end{pmatrix} = \begin{pmatrix} 1 & -1/\varepsilon \\ 0 & 1/\varepsilon \end{pmatrix}.$$

Then, the solution of the linear system $AX = B$, for the input data $B = (1, 1)^t$, is $X = (1-1/\varepsilon, 1/\varepsilon)^t$.

For approximate values of ε the previous linear system is solved by replacing matrix A with its corresponding approximate matrix $A_{approx} = \begin{pmatrix} 1 & 1 \\ 0 & \varepsilon_{approx} \end{pmatrix}$, where ε_{approx} is the truncation of ε to p digits. In this case, $det(A_{approx}) = \varepsilon_{approx}$. Tables 1 and 2 show the errors in the solution of the system $AX = B$ when ε is truncated to p digits.

Table 1: Numeric results by truncating ε to too few decimal digits.

ε	$(1/3) \times 10^{-1}$	$(1/3) \times 10^{-2}$
Truncation digits	2 digits	3 digits
ε_{approx}	3×10^{-1}	3×10^{-2}
cond(A)	60.0167	600.0017
cond(A_{approx})	66.6817	666.6682
$Inv(A)$	$\begin{pmatrix} 1 & -30 \\ 0 & 30 \end{pmatrix}$	$\begin{pmatrix} 1 & -300 \\ 0 & 300 \end{pmatrix}$
$Inv(A_{approx})$	$\begin{pmatrix} 1 & -33.33 \\ 0 & 33.33 \end{pmatrix}$	$\begin{pmatrix} 1 & -333.333 \\ 0 & 333.333 \end{pmatrix}$
X_{exact}	$(-29, 30)^t$	$(-299, 300)^t$
X_{approx}	$(-32.3300, 33.3300)^t$	$(-332.3330, 333.3330)$
AE(X_{approx}, X_{exact})	4.7093	47.1400
RE(X_{approx}, X_{exact})	0.1129	0.1113
ε	$(1/3) \times 10^{-3}$	$(1/3) \times 10^{-4}$
Truncation digits	4 digits	5 digits
ε_{approx}	3×10^{-4}	3×10^{-5}
cond(A)	6.0000×10^3	6.0000×10^4
cond(A_{approx})	6.6667×10^3	6.6667×10^4
$Inv(A)$	$\begin{pmatrix} 1 & -3000 \\ 0 & 3000 \end{pmatrix}$	$\begin{pmatrix} 1 & -30000 \\ 0 & 30000 \end{pmatrix}$
$Inv(A_{approx})$	$\begin{pmatrix} 1 & -3.3333 \times 10^3 \\ 0 & 3.3333 \times 10^3 \end{pmatrix}$	$\begin{pmatrix} 1 & -3.3333 \times 10^4 \\ 0 & 3.3333 \times 10^4 \end{pmatrix}$
X_{exact}	$(-2999, 3000)^t$	$(-29999, 30000)^t$
X_{approx}	$(-3.3323 \times 10^3, 3.3333 \times 10^3)^t$	$(-3.3332 \times 10^4, 3.3333 \times 10^4)^t$
AE(X_{approx}, X_{exact})	471.4045	4.71405×10^3
RE(X_{approx}, X_{exact})	0.1111	0.1111

Table 2: Numeric results by truncating ε to too few decimal digits..

ε	$(1/3)\times 10^{-7}$	$(1/3)\times 10^{-15}$
Truncation digits	8 digits	16 digits
ε_{aprox}	3×10^{-8}	3×10^{-16}
cond(A)	6.0000×10^{7}	6.0000×10^{15}
cond(A_{approx})	6.6666×10^{7}	6.6666×10^{15}
$Inv(A)$	$\begin{pmatrix} 1 & -3.0000\times 10^{7} \\ 0 & 3.0000\times 10^{7} \end{pmatrix}$	$\begin{pmatrix} 1 & -3.0000\times 10^{15} \\ 0 & 3.0000\times 10^{15} \end{pmatrix}$
$Inv(A_{approx})$	$\begin{pmatrix} 1 & -3.3333\times 10^{7} \\ 0 & 3.3333\times 10^{7} \end{pmatrix}$	$\begin{pmatrix} 1 & -3.3333\times 10^{15} \\ 0 & 3.3333\times 10^{15} \end{pmatrix}$
X_{exact}	$(-2.9999\times 10^{7}, 3.0000\times 10^{7})^t$	$(-2.9999\times 10^{15}, 3.0000\times 10^{15})^t$
X_{approx}	$(-3.3333\times 10^{7}, 3.3333\times 10^{7})^t$	$(-3.3333\times 10^{15}, 3.3333\times 10^{15})^t$
AE(X_{approx}, X_{exact})	4.71404×10^{6}	4.71404×10^{14}
RE(X_{approx}, X_{exact})	0.1111	0.1111
ε	$(1/3)\times 10^{-31}$	$(1/3)\times 10^{-63}$
Truncation digits	32 digits	64 digits
ε_{approx}	3×10^{-32}	3×10^{-64}
cond(A)	6.0000×10^{31}	6.0000×10^{63}
cond(A_{approx})	6.6666×10^{31}	6.6666×10^{63}
$Inv(A)$	$\begin{pmatrix} 1 & -3.0000\times 10^{31} \\ 0 & 3.0000\times 10^{31} \end{pmatrix}$	$\begin{pmatrix} 1 & -3.0000\times 10^{63} \\ 0 & 3.0000\times 10^{63} \end{pmatrix}$
$Inv(A_{approx})$	$\begin{pmatrix} 1 & -3.3333\times 10^{31} \\ 0 & 3.3333\times 10^{31} \end{pmatrix}$	$\begin{pmatrix} 1 & -3.3333\times 10^{63} \\ 0 & 3.3333\times 10^{63} \end{pmatrix}$
X_{exact}	$(-3.0000\times 10^{31}, 3.0000\times 10^{31})^t$	$(-3.0000\times 10^{63}, 3.0000\times 10^{63})^t$
X_{approx}	$(-3.3333\times 10^{31}, 3.3333\times 10^{31})^t$	$(-3.3333\times 10^{63}, 3.3333\times 10^{63})^t$
AE(X_{approx}, X_{exact})	4.71404×10^{30}	4.71404×10^{62}
RE(X_{approx}, X_{exact})	0.1111	0.1111

Table 3, considers the solution of the system of equations $AX = B$, when $\varepsilon = (1/3)\times 10^{-4}$, whose exact solution is $X_{exact} = (-29999, 30000)^t$. In this case, we observe that when we increase precision of ε to more digits the approximate solution X_{approx} is getting closer to the exact solution X_{exact}.

Table 3: Numeric results by increasing precision of $\varepsilon = (1/3) \times 10^{-4}$ to an adequate number of decimal digits.

Truncation digits	5 digits	6 digits
ε_{approx}	3×10^{-5}	33×10^{-6}
cond(A)	6.0000×10^4	6.0000×10^4
cond(A_{approx})	6.6667×10^4	6.0606×10^4
$Inv(A)$	$\begin{pmatrix} 1 & -30000 \\ 0 & 30000 \end{pmatrix}$	$\begin{pmatrix} 1 & -30000 \\ 0 & 30000 \end{pmatrix}$
$Inv(A_{approx})$	$\begin{pmatrix} 1 & -3.3333 \times 10^4 \\ 0 & 3.3333 \times 10^4 \end{pmatrix}$	$\begin{pmatrix} 1 & -3.0303 \times 10^4 \\ 0 & 3.0303 \times 10^4 \end{pmatrix}$
X_{exact}	$(-29999, 30000)^t$	$(-29999, 30000)^t$
X_{approx}	$(-3.3332 \times 10^4, 3.3333 \times 10^4)^t$	$(-3.0302 \times 10^4, 3.0303 \times 10^4)^t$
AE(X_{approx}, X_{exact})	4.71404×10^3	428.5496
RE(X_{approx}, X_{exact})	0.1111	0.0101
Truncation digits	7 digits	8 digits
ε_{approx}	333×10^{-7}	3333×10^{-8}
cond(A)	6.0000×10^4	6.0000×10^4
cond(A_{approx})	6.0060×10^4	6.0006×10^4
$Inv(A)$	$\begin{pmatrix} 1 & -30000 \\ 0 & 30000 \end{pmatrix}$	$\begin{pmatrix} 1 & -30000 \\ 0 & 30000 \end{pmatrix}$
$Inv(A_{approx})$	$\begin{pmatrix} 1 & -3.0030 \times 10^4 \\ 0 & 3.0030 \times 10^4 \end{pmatrix}$	$\begin{pmatrix} 1 & -3.0003 \times 10^4 \\ 0 & 3.0003 \times 10^4 \end{pmatrix}$
X_{exact}	$(-29999, 30000)^t$	$(-29999, 30000)^t$
X_{approx}	$(-3.0029 \times 10^4, 3.0030 \times 10^4)^t$	$(-3.0002 \times 10^4, 3.0003 \times 10^4)^t$
AE(X_{approx}, X_{exact})	42.4689	4.2431
RE(X_{approx}, X_{exact})	0.0010	1.0001×10^{-4}

ROUNDING ERRORS: In this case, we illustrate the errors obtained by rounding ε to n digits. Considering the same matrix of the previous example: $A = \begin{pmatrix} 1 & 1 \\ 0 & \varepsilon \end{pmatrix}$, where ε takes different positive small values, where $det(A) = \varepsilon$, and the inverse matrix of A is given by $A^{-1} = \begin{pmatrix} 1 & -1/\varepsilon \\ 0 & 1/\varepsilon \end{pmatrix}$, once again, the solution of the linear system $AX = B$ is $X = (1 - 1/\varepsilon, 1/\varepsilon)^t$, for the input data $B = (1, 1)^t$. The corresponding approximation matrix of A is given by $A_{approx} = \begin{pmatrix} 1 & 1 \\ 0 & \varepsilon_{approx} \end{pmatrix}$, where ε_{approx} is the rounding of ε to p digits, and $det(A_{approx}) = \varepsilon_{aprox}$. Tables 4 and 5 show the errors in the solution of the system of equations $AX = B$ when ε is rounded to p digits.

Table 4: Numeric results by rounding ε to too few decimal digits.

ε	$(2/3)\times 10^{-1}$	$(2/3)\times 10^{-2}$
Rounding digits	2 digits	3 digits
ε_{approx}	0.07	0.007
cond(A)	30.0334	300.0033
cond(A_{approx})	28.6065	285.7178
$Inv(A)$	$\begin{pmatrix} 1 & -15 \\ 0 & 15 \end{pmatrix}$	$\begin{pmatrix} 1 & -150 \\ 0 & 150 \end{pmatrix}$
$Inv(A_{approx})$	$\begin{pmatrix} 1 & -14.2900 \\ 0 & 14.2900 \end{pmatrix}$	$\begin{pmatrix} 1 & -142.8570 \\ 0 & 142.8570 \end{pmatrix}$
X_{exact}	$(-14, 15)^t$	$(-149, 150)^t$
X_{approx}	$(-13.2900, 14.2900)^t$	$(-141.8570, 142.8570)$
AE(X_{approx}, X_{exact})	1.0041	10.1017
RE(X_{approx}, X_{exact})	0.0489	0.0478
ε	$(2/3)\times 10^{-3}$	$(2/3)\times 10^{-4}$
Rounding digits	4 digits	5 digits
ε_{approx}	7×10^{-4}	7×10^{-5}
cond(A)	3.0000×10^{3}	3.0000×10^{4}
cond(A_{approx})	2.8571×10^{3}	2.8571×10^{4}
$Inv(A)$	$\begin{pmatrix} 1 & -1500 \\ 0 & 1500 \end{pmatrix}$	$\begin{pmatrix} 1 & -15000 \\ 0 & 15000 \end{pmatrix}$
$Inv(A_{approx})$	$\begin{pmatrix} 1 & -1.4286\times 10^{3} \\ 0 & 1.4286\times 10^{3} \end{pmatrix}$	$\begin{pmatrix} 1 & -1.4286\times 10^{4} \\ 0 & 1.4286\times 10^{4} \end{pmatrix}$
X_{exact}	$(-1499, 1500)^t$	$(-14999, 15000)^t$
X_{approx}	$(-1.4276\times 10^{3}, 1.4286\times 10^{3})^t$	$(-1.4285\times 10^{4}, 1.4286\times 10^{4})^t$
AE(X_{approx}, X_{exact})	101.0153	1.0102×10^{3}
RE(X_{approx}, X_{exact})	0.0476	0.0476

Table 5: Numeric results by rounding ε to too few decimal digits.

ε	$(2/3) \times 10^{-7}$	$(2/3) \times 10^{-15}$
Rounding digits	8 digits	16 digits
ε_{aprox}	7×10^{-8}	7×10^{-34}
cond(A)	3.0000×10^{7}	3.0000×10^{15}
cond(A_{approx})	2.8571×10^{7}	2.8571×10^{15}
$Inv(A)$	$\begin{pmatrix} 1 & -1.5000 \times 10^{7} \\ 0 & 1.5000 \times 10^{7} \end{pmatrix}$	$\begin{pmatrix} 1 & -1.5000 \times 10^{15} \\ 0 & 1.5000 \times 10^{15} \end{pmatrix}$
$Inv(A_{approx})$	$\begin{pmatrix} 1 & -1.4286 \times 10^{7} \\ 0 & 1.4286 \times 10^{7} \end{pmatrix}$	$\begin{pmatrix} 1 & -1.4286 \times 10^{15} \\ 0 & 1.4286 \times 10^{15} \end{pmatrix}$
X_{exact}	$(-1.5000 \times 10^{7}, 1.5000 \times 10^{7})^t$	$(-1.5000 \times 10^{15}, 1.5000 \times 10^{15})^t$
X_{approx}	$(-1.4286 \times 10^{7}, 1.4286 \times 10^{7})^t$	$(-1.4286 \times 10^{15}, 1.4286 \times 10^{15})^t$
AE(X_{approx}, X_{exact})	1.0102×10^{6}	1.0102×10^{14}
RE(X_{approx}, X_{exact})	0.0476	0.0476
ε	$(2/3) \times 10^{-31}$	$(2/3) \times 10^{-63}$
Rounding digits	32 digits	64 digits
ε_{aprox}	7×10^{-32}	7×10^{-64}
cond(A)	3.0000×10^{31}	3.0000×10^{63}
cond(A_{approx})	2.8571×10^{31}	2.8571×10^{63}
$Inv(A)$	$\begin{pmatrix} 1 & -1.5000 \times 10^{31} \\ 0 & 1.5000 \times 10^{31} \end{pmatrix}$	$\begin{pmatrix} 1 & -1.5000 \times 10^{63} \\ 0 & 1.5000 \times 10^{63} \end{pmatrix}$
$Inv(A_{approx})$	$\begin{pmatrix} 1 & -1.4286 \times 10^{31} \\ 0 & 1.4286 \times 10^{31} \end{pmatrix}$	$\begin{pmatrix} 1 & -1.4286 \times 10^{63} \\ 0 & 1.4286 \times 10^{63} \end{pmatrix}$
X_{exact}	$(-1.5000 \times 10^{31}, 1.5000 \times 10^{31})^t$	$(-1.5000 \times 10^{63}, 1.5000 \times 10^{63})^t$
X_{approx}	$(-1.4286 \times 10^{31}, 1.4286 \times 10^{31})^t$	$(-1.4286 \times 10^{63}, 1.4286 \times 10^{63})^t$
AE(X_{approx}, X_{exact})	1.0102×10^{30}	1.0102×10^{62}
RE(X_{approx}, X_{exact})	0.0476	0.0476

Table 6 shows the errors produced by rounding. Here, we consider the solution of the system of equations $AX = B$, when $\varepsilon = (2/3) \times 10^{-4}$, whose exact solution is $X_{exact} = (-14999, 15000)^t$. We can observe that the approximate solution X_{approx} is getting closer to the exact solution X_{exact}, when we increase the precision of ε to more digits.

Table 6: Numeric results by increasing precision of $\varepsilon = (2/3) \times 10^{-4}$ to an adequate number of decimal digits.

Rounding digits	5 digits	6 digits
ε_{approx}	7×10^{-5}	67×10^{-6}
cond(A)	3.0000×10^4	3.0000×10^4
cond(A_{approx})	2.8571×10^4	2.9851×10^4
$Inv(A)$	$\begin{pmatrix} 1 & -15000 \\ 0 & 15000 \end{pmatrix}$	$\begin{pmatrix} 1 & -15000 \\ 0 & 15000 \end{pmatrix}$
$Inv(A_{approx})$	$\begin{pmatrix} 1 & -1.4286 \times 10^4 \\ 0 & 1.4286 \times 10^4 \end{pmatrix}$	$\begin{pmatrix} 1 & -1.4925 \times 10^4 \\ 0 & 1.4925 \times 10^4 \end{pmatrix}$
X_{exact}	$(-14999, 15000)^t$	$(-14999, 15000)^t$
X_{approx}	$(-1.4285 \times 10^4, 1.4286 \times 10^4)^t$	$(-1.4924 \times 10^4, 1.4925 \times 10^4)^t$
AE(X_{approx}, X_{exact})	1.0102×10^3	105.5383
RE(X_{approx}, X_{exact})	0.0476	0.0050
Rounding digits	7 digits	8 digits
ε_{approx}	667×10^{-7}	6667×10^{-8}
cond(A)	3.0000×10^4	3.0000×10^4
cond(A_{approx})	2.9985×10^4	2.9999×10^4
$Inv(A)$	$\begin{pmatrix} 1 & -15000 \\ 0 & 15000 \end{pmatrix}$	$\begin{pmatrix} 1 & -15000 \\ 0 & 15000 \end{pmatrix}$
$Inv(A_{approx})$	$\begin{pmatrix} 1 & -1.4993 \times 10^4 \\ 0 & 1.4993 \times 10^4 \end{pmatrix}$	$\begin{pmatrix} 1 & -1.4999 \times 10^4 \\ 0 & 1.4999 \times 10^4 \end{pmatrix}$
X_{exact}	$(-14999, 15000)^t$	$(-14999, 15000)^t$
X_{approx}	$(-1.4992 \times 10^4, 1.4993 \times 10^4)^t$	$(-1.4998 \times 10^4, 1.4999 \times 10^4)^t$
AE(X_{approx}, X_{exact})	10.6013	1.0606
RE(X_{approx}, X_{exact})	4.9977×10^{-4}	4.9999×10^{-5}
