# Supplementary material for: An FPGA-based analysis of trade-offs in the presence of ill-conditioning and different precision levels in computations
Source: PLoS One. 2020 Jun 19;15(6):e0234293. doi: 10.1371/journal.pone.0234293 (PMC7304599; doi:10.1371/journal.pone.0234293)
Supplement: Attachment — Submitted filename: Response_letter.pdf [file pone.0234293.s003.pdf]

March 03, 2020

PLOS ONE Editorial Board

Hereby, I, on behalf of the authors of the research article, previously entitled “A trade-off analysis in presence of ill-conditioning and different precisions associated with applications to real-life problems”, now renamed “An FPGA-based analysis of trade-offs in the presence of ill-conditioning and different precision levels in computations”, thank you for the opportunity of revising and improving this manuscript.

Observations:

- As requested, we submit a copy of the manuscript showing the changes. In the edited version of the manuscript, and as a way to facilitate change-tracking, the following format is used:
    a) The eliminated parts are shown in red color and strikethrough as ~~EXAMPLE_ERASED~~;
    b) The added parts are shown highlighted as EXAMPLE_ADDED.
- We submit as well a clean copy of the edited manuscript to facilitate its reading.
- Additionally, we attach this letter with these details to facilitate change-tracking.
- Finally, we submit the certificate of English editing, provided by Editage.

In the following, we explain the main changes we made in the manuscript, which were made taking into account the comments made by the ***PLOS ONE*** Editorial Board:

1. Concerning the Introduction, we reframed it to focus more on our specific study on matrices. For that reason, we eliminated the mention referring to the Apollo mission issue from the first paragraph of the Introduction. Other additional changes, mentioned next, contribute to this reframing.
2. Regarding the comment about the need of having a clear definition of the research question, we added in lines 84-96 of the clean copy (lines 99-111 of the change-tracking copy) the research question, along with a brief explanation on how we test this question, with respect to the state-of-the-art methods.
3. Also, regarding the comparison with other state-of-the-art methods, we added *Table 6* to the **Discussion** section (**Comparisons** subsection), in order to simplify the reading of the differences with the other five implementations for matrix inversion, used for comparison purposes.
4. Finally, regarding the comments made by the editors about the grammar and overall readability of the manuscript, as suggested, we decided to resort to an editing service to properly copyedit the document, in order to satisfy the high-quality standards of ***PLOS ONE***.

Additionally, after a series of review rounds and discussions on the manuscript, and taking into account the suggestions made by the **Editage** editor as well as ours, we decided to add the following parts, expecting they contribute to strengthening the arguments presented:

1. As we mentioned at the beginning of this letter, the manuscript was renamed “An FPGA-based analysis of trade-offs in the presence of ill-conditioning and different precision levels in computations”, in an effort to make it more concise.

2. We provide more details concerning the important problems that arise when dealing with precision in computations, in lines 68-78 of the clean copy (lines 81-93 of the change-tracking copy).
3. Regarding the main contributions of the work, we added a third contribution, which connects the first two. This text is presented in lines 108-117 of the clean copy (lines 128-137 of the change-tracking copy).
4. We also added the outline of how the paper is organized, which is presented in lines 120-124 of the clean copy (lines 141-145 of the change-tracking copy).
5. In lines 252-263 of the clean copy (lines 282-296 of the change-tracking copy), we make an effort to clarify more why the matrix size and the specific matrix inversion method were chosen for the implementations.
6. After presenting and explaining Case study 1, we added some comments to emphasize the relevance of the results of this case, with respect to how ill-conditioning can affect. These changes appear in lines 377-381 of the clean copy (lines 422-426 of the change-tracking copy).
7. Likewise, to explain Case study 2, we added some comments to emphasize the fact that, although increasing precision might solve problems in calculations, it can be a problem by itself in terms of resource consumption. These changes are shown in lines 395-400 of the clean copy (lines 440-445 of the change-tracking copy).
8. To make more evident the growth factor in the use of resources of the implementations, and to present this information in a more organized form, we added *Table 5* to the **Results** section (**Trade-off analysis: hardware implementations and performance** subsection).
9. The works presented in the **Discussion** section (**Comparisons** subsection) were reorganized in order to present them in a chronological fashion.
10. To clarify how inadequate handling of precision can affect, we also added some lines to the Conclusions in lines 605-609 of the clean copy (682-686 of the change-tracking copy).
11. As suggested by the **Editage** editor, we added certain clarifications throughout the manuscript, to avoid ambiguity in several expressions.
12. Certain words and expressions were changed or eliminated after proof-reading the manuscript, which is shown following the same format specified at the beginning of this letter.
13. Finally, given that the mention of the Apollo mission issue was removed, the corresponding entry was removed from the **References** (Bennett, 1970).

We hope that, with the changes made in the manuscript, we have properly addressed your concerns so its revised version can be considered for its publication in ***PLOS ONE***.

Best regards,

Carlos Arturo Hernández-Gracidas

CONACYT-BUAP
